# Reproductive development and genetic structure of the mycoheterotrophic orchid *Pogoniopsis schenckii* Cogn.

**DOI:** 10.1186/s12870-021-03118-y

**Published:** 2021-07-12

**Authors:** Mariana Ferreira Alves, Fabio Pinheiro, Carlos Eduardo Pereira Nunes, Francisco Prosdocimi, Deise Schroder Sarzi, Carolina Furtado, Juliana Lischka Sampaio Mayer

**Affiliations:** 1 Departamento de Biologia Vegetal, Instituto de Biologia, Universidade Estadual de Campinas, São Paulo, Brazil; 2 Laboratório de Genômica e Biodiversidade, Instituto de Bioquímica Médica Leopoldo de Meis, Universidade Federal Do Rio de Janeiro, Rio de Janeiro, Brazil; 3 Departamento de Genética, Instituto Nacional de Câncer, Rio de Janeiro, Brazil

**Keywords:** Autogamy, Embryology, Fungal hyphae, Genetic diversity, Mycoheterotrophy, Orchid

## Abstract

**Background:**

*Pogoniopsis schenckii* Cogn. is a mycoheterotrophic orchid that can be used as a model to understand the influence of mycoheterotrophy at different stages of the reproductive cycle. We aimed to verify the presence of endophytic and epiphytic fungi at each stage of the reproductive process and investigated how the breeding system may relate to genetic structure and diversity of populations. In this study we performed anatomical and ultrastructural analyses of the reproductive organs, field tests to confirm the breeding system, and molecular analysis to assess genetic diversity and structure of populations.

**Results:**

During the development of the pollen grain, embryo sac and embryogenesis, no fungal infestation was observed. The presence of endophytic fungal hyphae was observed just within floral stems and indehiscent fruit. Beyond assuring the presence of fungus that promote seed germination, specific fungi hyphae in the fruit may affect other process, such as fruit ripening. As other mycoheterotrophic orchids, *P. schenckii* is autogamous, which may explain the low genetic diversity and high genetic structure in populations.

**Conclusions:**

We discuss an interesting interaction: fungal hyphae in the indehiscent fruit. These fungal hyphae seem to play different roles inside fruit tissues, such as acting in the fruit maturation process and increasing the proximity between fungi and plant seeds even before dispersion occurs. As other mycoheterotrophic orchids, *P. schenckii* is autogamous, which may explain the low genetic diversity and high genetic structure in populations. Altogether, our findings provide important novel information about the mechanisms shaping ecology and evolution of fragmented populations of mycoheterotrophic plant.

**Supplementary Information:**

The online version contains supplementary material available at 10.1186/s12870-021-03118-y.

## Background

Mycoheterotrophy is the parasitic relationship between plants and fungi in which the plants depend on the interaction with fungi to fulfill part of their nutritional needs [1]. In presenting an unconventional way of life, mycoheterotrophic plants have been arousing botanical curiosity for many years [2]. Mycoheterotrophy has independently evolved in several lineages, being reported in 10 angiosperm families and approximately 515 species [3]. These plants have lost their photosynthetic capacity and rely on carbon from associations with fungi throughout their whole life cycle [2]. Due to their dependence on an obligate interaction, these species have a restricted distribution, usually smaller than the distribution of the exploited fungi [4]. They are mostly found in low-light locations of underbrushes, where photosynthetic plants would be at a competitive disadvantage [1].

Mycoheterotrophic plants exhibit numerous modifications. They are small, herbaceous, with reduced vegetative organs and, for most of their life cycle, remain underground [2]. Infestations with fungi are not only found in the roots, but also in underground organs such as rhizomes and corms. Fungal infections in aerial organs have not been described thus far, indicating that fungi propagation in the plant seems to be restricted to organs close to the soil [2]. In seeds, infection generally occurs in a short period after its germination, but in some cases, such as for mycoheterotrophic orchids, fungal infection is mandatory for seed germination [1, 2, 5, 6]. The first studies on mycoheterotrophic species were focused on the relationship between plant and fungi, though data on development, reproductive biology, genetics and ecology have drawn attention recently [1–3].

Due to high levels of specialization found in mycoheterotrophic species, most of which are related to reductions in morphological traits [2] and genomes [7]; the association with fungal species is considered obligate. Accordingly, Bidartondo [1], proposed that it would be evolutionarily unstable for a mycoheterotrophic species already involved in an obligate symbiotic interaction to establish additional obligate associations. Thus, these species are expected to present reproductive strategies that are less dependent on biotic vectors such as a generalist pollination, spontaneous self-pollination and/or allocation of resources for seed formation.

How mycoheterotrophy can impact the reproduction of these species is still a debatable issue. Autogamy is, in fact, observed in many mycoheterotrophic plants [8–13]. However, species with mixed pollination systems can be observed, such as those with self- and cross-pollination mechanisms [14–17], but we can also find completely xenogamous species, in which reproductive success depends on insect pollination [8]. Considering that mycoheterotrophy influences the reproductive system of species, we should also expect reflections in the genetic structure of these populations. Indeed, genetic population studies revealed low levels of diversity and interpopulation gene exchange in mycoheterotrophic species, suggesting a prevalence of autogamy [18–22].

Orchidaceae, one of the most diverse families of angiosperms, accounts for about 235 mycoheterotrophic species [3]. Mycoheterotrophy has evolved independently several times within the family [23], suggesting convergent functional evolution, achieved through a widespread absence of morphological and physiological constraints associated with photosynthetic apparatus loss. Although Orchidaceae comprises 45% of the mycoheterotrophic species known for angiosperms, there are a few studies integrating multidisciplinary approaches which aim to understand the ecological and evolutionary consequences of mycoheterotrophy (but see [19, 24, 25]). *Pogoniopsis* is a Brazilian endemic genus composed of two mycoheterotrophic species. The reproductive data for the genus show the absence of floral rewards and reproduction by autogamy [26], however, details of each reproductive stage and how this autogamy impacts the genetic population diversity have not been investigated. Another study reported the presence of fungi in the floral stem and fruits of *P. schenckii* Cogn. [27], differing from what has been described for other species, where the interaction only occurs underground [2]. In this study conducted by Sisti et al. [27], the genera of fungi found on the floral stem and fruits of the species were identified, nevertheless, the effects that this interaction has on the life cycle of the species was not evaluated. Thus, *Pogoniopsis* may be used as a model to understand structural, ecological and evolutionary consequences of mycoheterotrophy on reproductive stages and dynamics of the species. Therefore, we answer the following questions: (a) What is the structural pattern of fungal colonization of the fruits of *P. schenckii*? (b) Does interaction with fungal hyphae occur during the anther, ovule and embryo development? (c) Is autogamy the predominant breeding system of this species, and to what extent limitations in seed and pollen movement shape the genetic structure in this rare and endemic orchid species? Finally, we discuss how fungal colonization may influence the development of reproductive organs in this plant and how the breeding system and pollination interactions may influence the genetic structure of this rare orchid.

## Results

### Anther structure, microsporogenesis, microgametogenesis and germination of pollen grain

*Pogoniopsis schenckii* presents tetrasporangiate anther (Supplementary file Fig. S1a). In the initial stages, the anther primordium is composed of meristematic tissue. In young bud stamens, the anther wall is formed by epidermis, endothecium, middle layer, and tapetum, all uniseriate. The epidermis and endothecium are formed by periclinal flattened cells and remain intact throughout the development of the male gametophyte. The middle layer has compressed cells that are reabsorbed throughout development. The tapetum is glandular and consists of dense cytoplasm and evident nucleus cells. During the process of male gametophyte formation, tapetum cells are absorbed.

In the young anther, sporogenous cells differ as microspore mother cell (MiMC), which present evident nucleus. The first phase of the meiotic division of the MiMc, originates a dyad of cells (Supplementary file Fig. S1b, c). After this phase, we can observe cell wall formation. Dyad cells undergo the second stage of meiosis, originating a tetrad of microspores, which remain united and have different formats (Supplementary file Fig. S1d).

Subsequently, microgametogenesis occurs, and microspores undergo asymmetric cell division, and after cytokinesis, they originate pollen grains. The male gametophyte is formed by a bigger, vegetative cell, and a smaller, generative cell (Supplementary file Fig. S1e). In newly opened flowers, the anther presents ruptured epidermis, and the ripe pollen grain is exposed (Supplementary file Fig. S1f-h). Pollen grains germinate within the pollinia, and their tubes grow toward the stigma promoting self-pollination. The pollen tube takes about eight days to reach the ovary (Supplementary file Fig. S1h). During the development of pollen grain no evidence of fungal infestation was observed.

### Ovule development, megasporogenesis, megagametogenesis and embryogenesis

Soon after the beginning of pollen tube growth, placental proliferation occurs due to intense mitotic activity (Supplementary file Fig. S2a). Differentiation of ovules begins two days after spontaneous self-pollination. In the ovules primordia, a cell from the subepidermal layer differentiates into an archesporial cell, which directly originates the megaspore mother cell (MMC), increasing its volume and presenting an evident nucleus (Supplementary file Fig. S2b). The first stage of the meiotic division of the MMC givens rise to a dyad of megaspores (Supplementary file Fig. S2b, d). Only the chalazal cell of the dyad undergoes the second stage of meiosis, originating a triad of megaspores. The chalazal megaspore becomes functional, whereas the other megaspores degenerate (Supplementary file Fig. S2e).

After the first mitosis of the megagametogenesis, the binucleate megagametophyte showed several small vacuoles in the cytoplasm. Subsequently, a large central vacuole was formed between the nuclei and each nucleus moved into one pole of the megagametophyte (Supplementary file Fig. S2f). The two nuclei of the megagametophyte undergo the second cycle of mitotic divisions, forming the tetranucleated stage (Supplementary file Fig. S2g, h). The four nuclei undergo the third and last division, originating a megagametophyte with eight nuclei.

During the megagametophyte cellularization, the two synergids and egg cell are organized, most often in a triangular arrangement, to form the egg cell apparatus. Two nuclei migrate to the center of the megagametophyte and are now identified as polar nuclei. The three remaining nuclei in the chalazal region degenerate and do not form antipodes. The species ovule is tenuinucellate and ategmic (Supplementary file Fig. S2i). Fertilization occurs about 25 days after floral opening and can be evidenced by densely colored cytoplasm and zygote formation (Supplementary file Fig. S3a, b). Fusion was observed between polar nuclei and gametic nucleus; however, the primary nucleus of the endosperm is not divided and absorbed. The zygote undergoes an asymmetric transverse division, generating an apical and a basal cell (Supplementary file Fig. S3c). The basal cell is not divided and forms the suspensor. The apical cell undergoes more transverse and longitudinal divisions, originating embryos with a maximum of six cells (Supplementary file Fig. S3d-f). Protein substances and starch grains were observed in the mature embryo (Supplementary file Fig. S3g, h). During the development of embryo sac and embryogenesis no evidence of fungal infestation was observed.

### Morphology of the developing fruit

The fruit of *P. schenckii* has yellow coloration, and its development stems from the moment of the floral opening to the damping off of the fruit on the soil, a period that lasts about four months (Supplementary file Fig. S4a-d). About six days after floral opening, the stretching of the inferior ovary region starts. This expansion continues for about three months after floral opening (Supplementary file Fig. S4a). The fruit of approximately three months begins to present a darker coloration, and four months after floral opening it is completely dark (Supplementary file Fig. S4b-d). Then, there is the damping off of the floral stem and the fruit on the soil. After the fruit decomposes, the seeds are released into the soil.

### Fruit ontogenesis

**Fig. 1:**
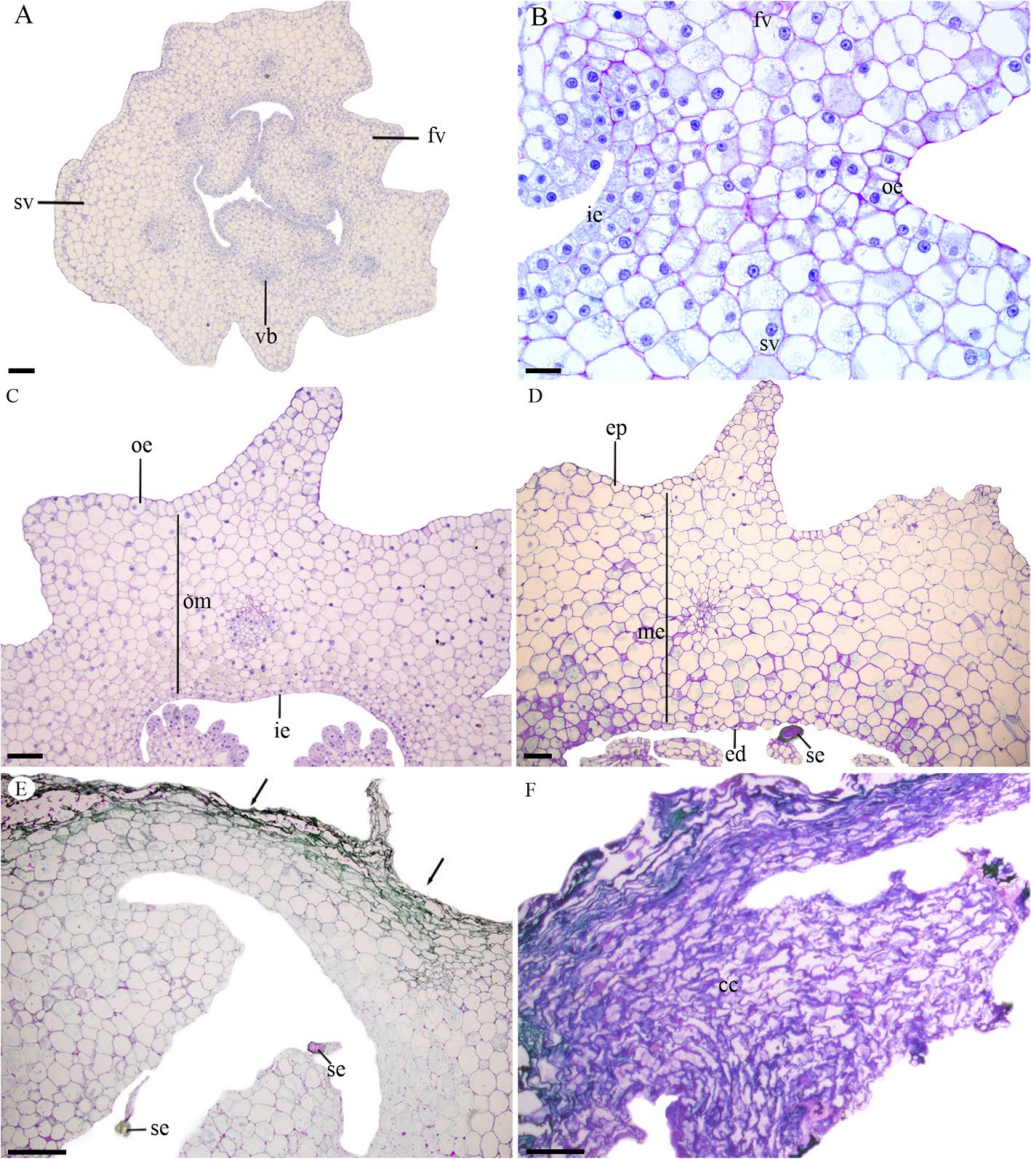
Longitudinal sections of *Pogoniopsis schenckii* fruits. **A** General view of the ovary, showing the fertile and sterile valves. **B** Detail of the fertile and sterile valve, where no dehiscence line is formed. **C** Median region of the ovary. **D** Median region of a young fruit. **E** Ripe fruit, with collapsed cells. Arrows indicate the cell collapse of the epicarp towards the endocarp. **F** Detail of the placental region of a ripe fruit with collapsed cells. cc = collapsed cells; ie = inner epiderms; ed = endocarp; ep = epicarp; fv = fertile valve; oe = outer epiderms; om = ovarian mesophyll; me = mesocarp; se = seed sv = sterile valve; vb = vascular bundle. Scale bars: 50 µm

The anthetic flower of *Pogoniopsis schenckii* presents inferior, unilocular ovary composed of three carpels and six valves, namely three fertile valves in the placenta region and three sterile valves (Fig. 1a). The outer and inner epidermis of the ovary is uniseriate (Fig. 1b). The ovarian mesophyll is composed of parenchymatic cells, and the cells near the external epidermis are bigger when compared with the cells of the internal epidermis (Fig. 1c). A single vascular bundle was observed in each of the valves, and in all valves, the vascular bundles are close to the inner epidermis (Fig. 1a). In the fertile valves, the placental region remains undifferentiated, only presenting projections (Fig. 1c).

After pollination, there is the proliferation of placental cells in each fertile valve, and in the apex of projections there is the formation of the ovule. Some anatomical changes occur in the pericarp of the fruit during its development (Fig. 1a-f). In the ovary, in the sterile valves we can find about 13 layers of parenchymatic cells (Fig. 1c), where anticlinal divisions occur for promoting growth in the fruit diameter. In the young fruit, in the sterile valves are about 15 layers of parenchymatic cells (Fig. 1d), and the increase in cell volume is the main factor responsible for fruit growth.

**Fig. 2:**
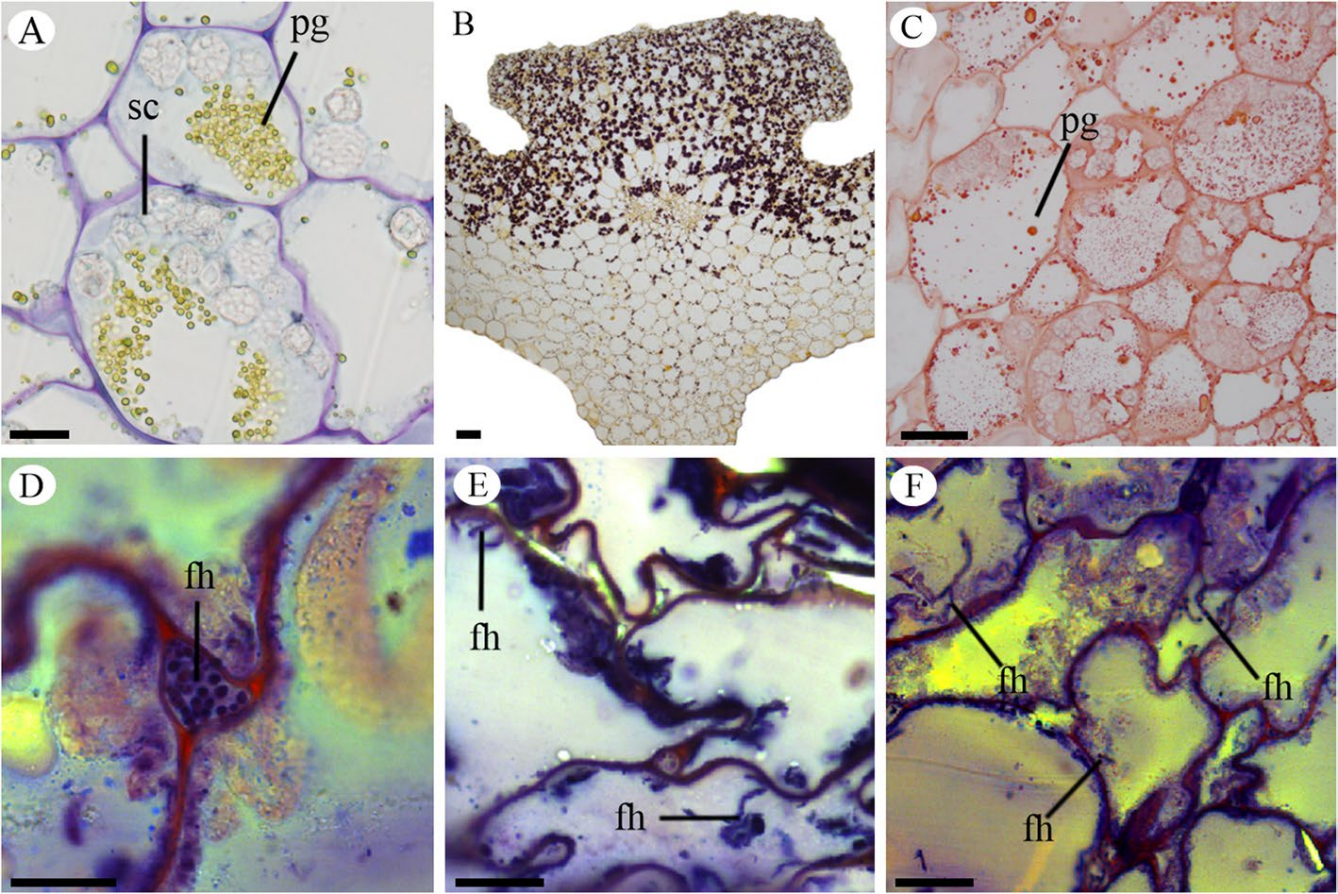
Longitudinal sections of the *Pogoniopsis schenckii* fruit. **A** Sterile valve with starch grains and protein granules evidenced by Toluinine Blue. **B** Placental region showing starch grains evidenced by Lugol. **C** Region placenta showing protein granules evidenced by Xylidine. Detail of the fruit showing protein granules. **D-F** Presence of fungal hyphae in the placental region evidenced by double staining with cotton blue and safranin. **D** Fungal hyphae between cells. **E–F** Fungal hyphae inside cells. fh = fungal hyphae; pg = protein granules; sc = starch grains. Scale bars: 50 µm

**Fig. 3:**
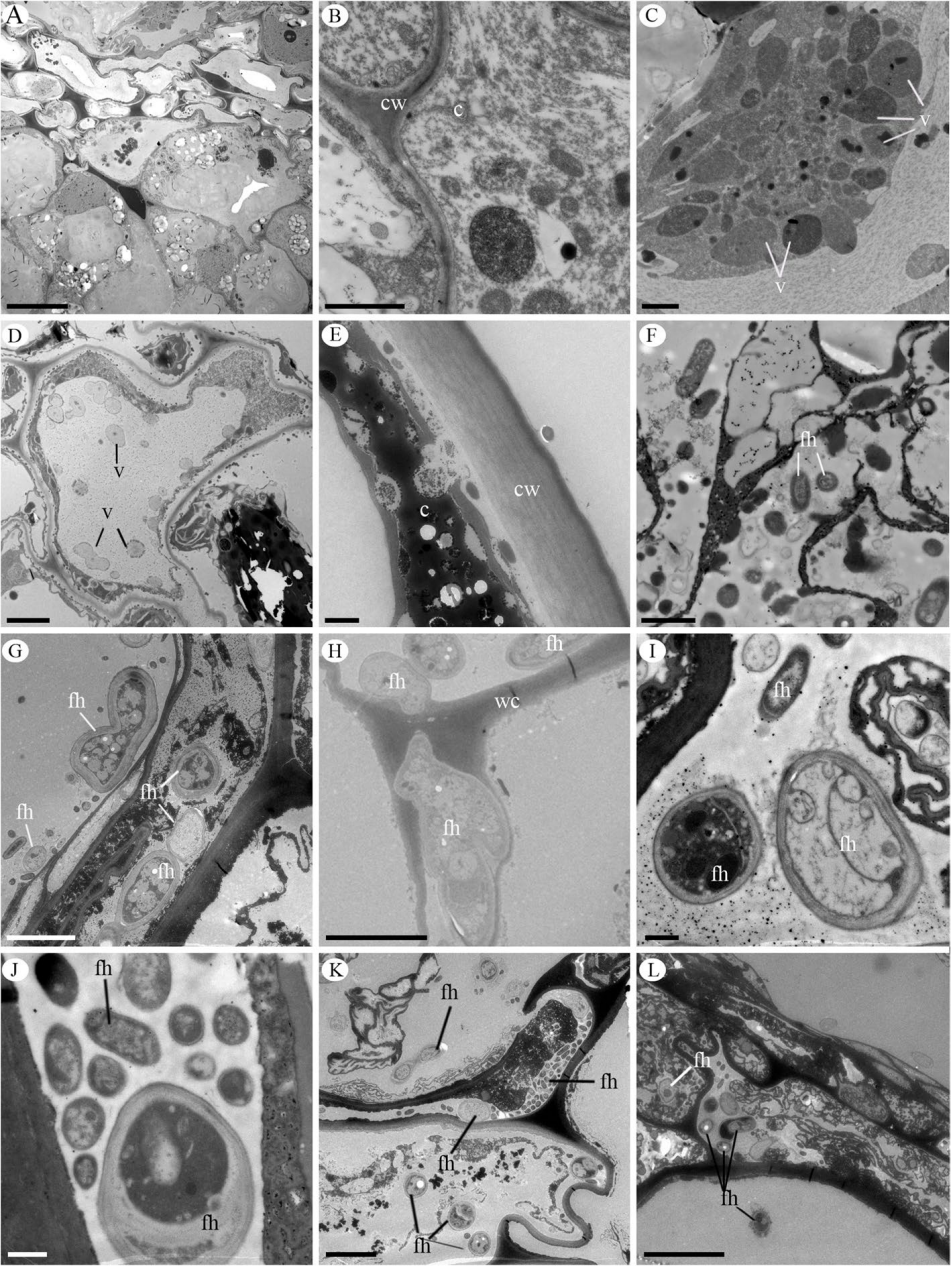
Transmission microscopy electromicrograph in the mesocarp and placental region of *Pogoniopsis shenckii*. **A** Overview of fruit cells with high cytoplasmic content. **B** Detail of the dense cytoplasm. **C** Vesicles concentrated in the cytoplasm. **D** Vesicles scattered in the cell vacuole. **E** Dense cytoplasm, release of secretion near the cell wall. **F-L** Fungal hyphae. **F** Fungal hyphae in the cell of the placental region. **G** Fungal hyphae in the mesocarp cells. **H** Fungal hyphae penetrating the cell wall. **I-J** Detail of the fungal hyphae in the mesocarp cells. **K** Fungal hyphae in the mesocarp cells of the ripe fruit. **L** Fungal hyphae in the collapsed cells of the ripe fruit. c = cytoplasm; fh = fungal hyphae; cw = cell wall; v = vesicles. Scale bars: I,J = 500 nm, E,F = 1 µm, B,C = 2 µm, D,G,H,K = 5 µm, A = 20 µm, L = 500 µm

The parenchymatic layers of the mesocarp of the *P. schenckii* fruit present cells with different contents (Figs. 2a-f and 3a, b). We observed accumulation of starch grains and protein granules in the flower ovary and young fruit (Fig. 2a-c). In fertile valves, starch grains are found in the placental region, and in sterile valves they are always close to the endocarp cells (Fig. 2a, b). Protein granules are found in parenchymatic cells of the mesocarp and in the placental region (Fig. 2a, c). During fruit development, we could observe the presence of vesicles (Fig. 3c-e), which can be found grouped in the case of the cytoplasm (Fig. 3c), in the vacuole (Fig. 3d) or releasing their contents into the cell wall (Fig. 3e).

In the mature fruit, adjacent epicarp and mesocarp cells begin to collapse (Fig. 1e, f). This process takes place in the whole fruit, always from the epicarp towards the endocarp and, when the fruit is ripe, epicarp and mesocarp cells are collapsed, thus reducing the thickness of the pericarp (Fig. 1f). At this stage of development, the pericarp is dark (Supplementary file Fig. S4c, d). At this moment, the floral stem falls, leaving the fruits in the soil, and decomposition begins. We did not observe the formation of the dehiscence line in any of the developmental stages of the fruit, characterizing it as indehiscent. We also observed fungal hyphae in the innermost layers of the mesocarp and in the placenta of the *P. schenckii* fruit (Figs. 2d-f and 3f-l). Fungal hyphae occur within cells (Figs. 2d-f and 3g-i), and are able to cross the cell wall (Fig. 3h). In these infected cells, besides the fungal hyphae, we also observed the presence of vesicles in the cytoplasm and vacuole. These hyphae were observed from the second month of fruit development, being found in a greater quantity in the mature fruit (Fig. 3j-l) when all cells were already collapsed (Figs. 1f and 3k, l).

### Breeding system, diversity and genetic structure

All floral buds in the spontaneous self-pollination treatment, and all flowers in the natural pollination treatment developed into fruits. No floral reward was found on flowers. In fact, we did not observe the presence of any floral visitors in *P. schenckii* flowers during monitoring.

The number of alleles per locus (*A*) in the populations ranged from 20 to 29. The expected (*H*_E_) and observed (*H*_O_) heterozygosity per population ranged from 0.428 to 0.66, and from 0.197 to 0.328, respectively. The inbreeding coefficient (*f*) ranged from 0.42 to 0.54 (Table 1). Clonal plants were found in the three analyzed populations, being 4 clones in the population of Pirapitinga, 3 in the population of Poço do Pito, and 1 in São Lourenço. Most of clones originated from multiple genotypes. Pirapitinga consisted of the population with the lowest G/N ratio, Nei diversity (Div), and uniformity of the effective number of genotypes (Eve) (Table 1).

**Table 1:** Characterization of genetic variability and clonal diversity in *Pogoniopsis schenckii* populations were estimated from eight nuclear microsatellite loci for 79 individuals. The number of alleles (*A*), number of private alleles (*PA*), expected (*H*_E_) and observed (*H*_O_) heterozygosity and the within-population inbreeding coefficient (*f*), population size (*Num*), number of genotypes (*Gen*), total number of individuals in a population (G/N), effective number of genotypes (*Eve*), and Nei’s genetic diversity (*Div*)

Populations	A	PA	*H*E	*H*O	*f*	Num	Gen	G/N	Eve	Div
Pirapitinga	27	0,533	0,428	0,197	0,542	31	27	0,870	0,790	0,985
Poço do Pito	29	0,863	0,660	0,328	0,506	37	34	0,920	0,900	0,994
São Lourenço	20	0,533	0,466	0,272	0,427	11	10	0,910	0,930	0,982

The analysis of molecular variance (AMOVA) between populations showed high and significant (*P* < 0.001) differentiation, with global *F*ST and *D*ST values of 0.233 and 0.312, respectively. The Mantel test was not significant (*p* = 0.163), demonstrating that populations do not show isolation-by-distance. We identified three distinct genetic clusters (K = 3) corresponding to the analyzed populations in the assignment test performed with MAVERICK program (Supplementary file Fig. S5). Despite the identification of three genetic groups, we found admixed individuals among populations.

## Discussion

In this study, the analysis of reproductive organ development shows an unusual interaction in this mycoheterotrophic plant, the presence of fungal hyphae during the maturation of the indehiscent fruit, allow a proximity between fungi and seeds during the fruit maturation process. The absence of pollinator visits and high fruit set levels in bagged flowers indicate that fruits are formed by autogamy. Indeed, genetic markers demonstrated high levels of inbreeding and genetic structure, suggesting a prevalence of autogamy in all populations. These data inform us about the extensive plant/fungus association in reproductive tissues, it also contain important information about mechanisms that shape the genetic structure of small and fragmented populations of mycoheterotrophic plants.

### Anatomy of reproductive organs

The stages of microsporogenesis and microgametogenesis result in the formation of a viable pollen grain, with developmental patterns like other species in the family [28]. At the time of floral opening, *P. schenckii* presents the placental region only with ovule primordia. This is a common feature in Orchidaceae, in which ovule development only occurs after the pollination stimulus [29–32].

Processes of megasporogenesis and megagametogenesis generate an embryo sac with eight nuclei, of which five undergo cellularization, with *Polygonum-*type development. Reduction in the number of cells in the embryo sac has been described for several species of the family [33–39], and may be related to anomalies in processes of cell division or the rapid degeneration of the nuclei of the chalazal pole, as we observed in this study [28, 40, 41]*.* About one month after the spontaneous self-pollination process, there is fertilization and formation of an undifferentiated embryo and of a primary endosperm nucleus, which subsequently degenerates. These characteristics are common in Orchidaceae, but also for mycoheterotrophic species from different families. Simple embryos, without differentiation of the epityl-hypocotyl axis and the cotyledonary region, with reduced or absent endosperm, have been observed in mycoheterotrophic species of different families [2, 37, 39, 42].

*Pogoniopsis schenckii* presents an ovary composed of three carpels and six valves such as the other orchid species which have been studied so far [43–46]. The sterile valve corresponds to the bases of a carpel corresponding with the base of the sepal, and the fertile valve corresponds to two carpel-halves with the base of the petal [46]. In *P. schenckii,* there is an extreme fusion between the wall of the ovary and the hypanthium; in addition, the species presents ovaries and fruits with an irregular morphology, which hinders the delimitation of carpels and valves.

The increased volume of parenchymatic layers of the mesocarp was the main factor in the growth in diameter of the fruit, which has already been described for some orchids [47, 48]. The presence of sclerenchyma cells in the fruit mesocarp was described for Orchidaceae species that have been studied so far, with the exception of our research [39, 47, 49–53]. The absence of sclerification observed in *P. schenckii* may be related to the fruit being indehiscent and having no need to present a rigid mesocarp; in addition, it can facilitate the decomposition process, which is a necessary factor for the dispersion of seeds. Orchidaceae fruits are dehiscent capsules [46], however, the loss of the dehiscence line characterizes the fruit as indehiscent.

In mature indehiscent fruits, cell death in the mesocarp is common [54]. During the development of *P. schenckii* fruit, we observed secretory vesicles in the cytoplasm and vacuoles of the mesocarp cells, which may indicate the occurrence of programmed cell death. Vesicles may perform different functions, including carrying lytic enzymes which are responsible for degrading cellular material once they are released [55–57]. This process has been described in different stages such as fruit ripening, fruit drop [58], development of seed integument [59], and during flower senescence [57]. During the fruit ripening process, cell collapse always occurs from the epicarp towards the endocarp. This pattern observed in this species differs from what has been observed during the maturation of indehiscent fruits, where cell disintegration firstly occurs in the core cells of the carpel, as seen in species of Solanaceae and Cactaceae [54, 60].

### Interaction between fruit and fungal hyphae

**Fig. 4:**
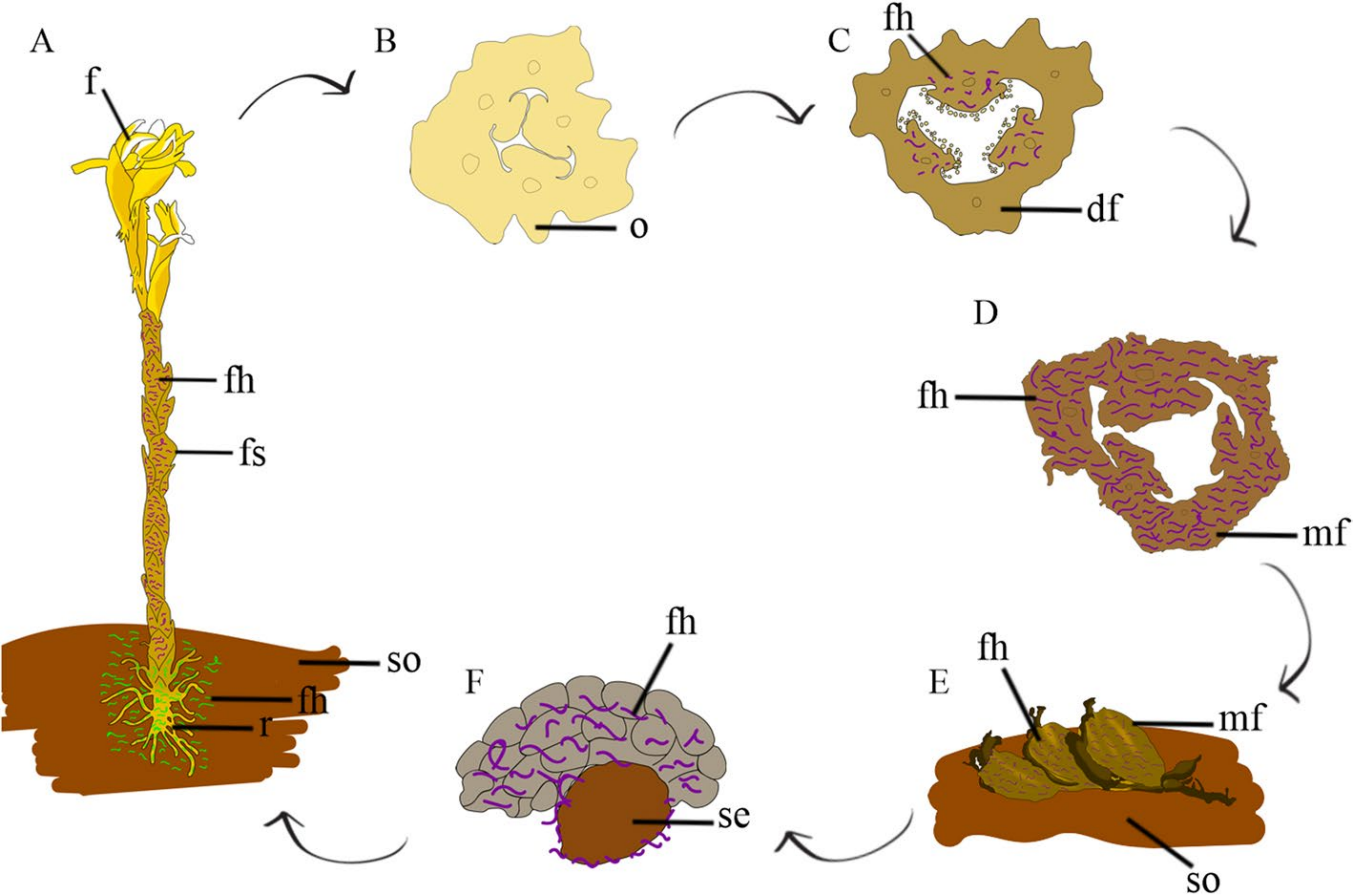
Schematic model of the participation of fungus hyphae over the life cycle of *Pogoniopsis schenckii.* **A** Individual showing different interactions with fungi. In purple, fungal hyphae that interact with the floral stem. In green fungal hyphae that interact with the roots. **B** Flower ovary. Note that has not interactions with fungal hyphae. **C** Fruit in development. At this stage, it is possible to observe interaction between the cells of the fruit and fungal hyphae in the placental region. Fungal hyphae are represented in purple. **D** Ripe fruit showing fungal hyphae. Fungal hyphae are represented in purple. **E** Ripe fruit dropped on the ground. Fungal hyphae are represented in purple. **F** Seed released after the fruit disintegrates. At this moment, it is already involved with the fungi that will promote its germination. Fungal hyphae are represented in purple. df = developing fruit; f = flower; fh = fungal hyphae; fs = floral stem; mf = mature fruit; o = ovary; r = root; se = seed; so = soil

The analyses showed that during the development of the anther, embryo sac and embryogenesis, no fungal infestation was observed, this type of interaction being restricted only to the floral stem and fruit (Fig. 4a-c). Fungal hyphae found on the floral stem grow and inoculate the fruit (Fig. 4a-e), and were observed throughout the fruit development process, being found in greater quantity on the ripe fruit (Fig. 4c-e). For this reason, they appear to have different functions in the life cycle of *P. schenckii*. They could act during the maturation of fruit, in the programmed cell death process, contributing to the process of cell disintegration and enhancing seed germination in the species.

Due to the nature of the interaction, fungi in the fruits are considered endophytic, as colonization occurs discreetly and is initially asymptomatic [61, 62]. However, endophytic fungi may become pathogenic during host senescence [63], a situation that seems to occur in the ripe fruit of the species. The fungi hyphae observed in the fruit have characteristics of hemibiotrophic fungi. Initially, they establish a food relationship without killing their hosts, but there is then a change in their lifestyle, killing the host cells, often by secreting toxins [64]. Thus, the secretory vesicles observed during the fruit maturation process in this species (Fig. 3c, d) may be a plant response to the presence of fungus within the tissue. The plant-pathogen interaction observed in the species indicates that the process of programmed cell death in the fruit occurs due to epigenetic reprogramming. Recent studies indicate that the gene expression of endophytic fungi can act as epigenetic modulators [65, 66], activating the expression of silent genes that can alter the profile of secondary metabolites of endophytic fungi, which triggers epigenetic changes in the host plant [67–69]. In this context, future studies should investigate which metabolites the fungi produce during fruit development, specifically which hormonal and genetic signaling may be involved in the process of programmed cell death in the developing fruit.

Orchid seeds are small with embryo and endosperm reduced, requiring association with fungi for their germination [2, 70]. For *P. schenckii* previous results show that the fungi found in the floral stem and fruit of the species belong three genera of the phylum Ascomycota—*Fusarium*, *Colletotrichum* and *Clonostachys*—[27]. In this study, fungi isolated from both fruit and root were inoculated into the seed of the species. Of these, fungus belonging to the genus *Fusarium*, *Clonostachys* and *Trichoderma*, were able to break the seed envelope, and *Clonostachys* was able to stimulate the development of the protocorm [27]. Some studies have suggested that for Orchidaceae, many specific fungal association and non-compatible mycorrhiza, may stimulate seed germination, however, will not support seedling development [71, 72]. This situation was observed in *P*. *schenckii*, since only one of the fungi that promote the rupture of the seed envelope was able to initiate the development of the protocorm [27]. Thus, for this mycohetrotrophic species, the fungal hyphae in the placental region can allow a proximity between fungi that could provide the initial stage of seed germination. In this way, it is possible these hyphae involve the seed and are dispersed next to it on the soil (Fig. 4e, f), favoring seed germination and initiation of protocorm development. Future studies might clarify which fungal associations are related to protocorm development and plantule establishment.

### Breeding system, diversity and genetic structure

*Pogoniopsis schenckii* presents sexual reproduction with self-fertilization, resulting from spontaneous self-pollination. Autogamy seems to be the predominant reproductive strategy in the species, since we observed no visitor/pollinator throughout the study. Our results corroborate the hypothesis proposed by Bidartondo [1], who believes that it would be evolutionarily unstable for a mycoheterotrophic species already involved in an obligate symbiotic relationship to establish additional ecological interactions, such as animal pollination. Thus, *P. schenckii* canalizes its energy resources for self-pollination, promoting seed production instead of floral rewards. This can be considered a reproductive assurance that allows plant reproduction independent of animal pollinators [73], as observed in this study.

Breeding systems are the greatest determinants for genetic variation in plants [74, 75]. For *P. schenckii,* there is low genetic variability and high inbreeding coefficient (*f*). The obtained data corroborates the low genetic variability that has been described in mycoheterotrophic orchids of *Corallorhiza* and *Gastrodia* species, in addition to high inbreeding coefficient for *Gastrodia* species [20–22]. These results can be explained by a set of factors such as the absence of pollinators associated with autogamy, low pollen flow, and restricted seed dispersion.

Low genetic diversity in rare and typically small species, such as *P. schenckii*, may result from loss of alleles, a consequence of genetic drift [18, 76]. Although *P. schenckii* presents low genetic variability, it can be deemed high when compared with other autogamous mycoheterotrophic species [20–22]. Proximately, this can be explained by rare cross-pollination events, or by the occurrence of mutations. In *Arabidopsis,* researchers observed that mutations may occur at higher frequencies in autogamous lineages, increasing values of genetic diversity [77], a process that may also occur in *P. schenckii.* Evolution in species that perform self-pollination may differ from species that perform cross-pollination. Endogamy can reduce the genetic diversity of populations, but it can also slightly increase the likelihood of dominant mutations [78, 79]. The number of clones observed in the population was low, which demonstrates that self-fertilization can provide reproductive assurance for this species.

The obtained results show a strong population structure (*F*_ST_ = 0.233 and *D*_ST_ = 0.312), and the presence of three genetic groups, which may be explained by the high rate of inbreeding and low pollen flow between populations. This pattern is commonly observed in mycoheterotrophic orchids [19, 21]. *Pogoniopsis schenckii* presents indehiscent fruit, which damp off on the ground when ripe, subsequently beginning decomposition. Thus, the germination process and the establishment of protocorm probably take place near the mother plant. This may be another process that, when associated with autogamy, reinforces the genetic structure of each population.

It is worth highlighting that mycoheterotrophic species are obligate dependents on fungal association for their survival [1, 2]. Therefore, its populations are naturally fragmented, since their distribution depends of the occurrence of their mycorrhizal partners [2, 80]. Moreover, the monitoring of *P. schenckii* populations shows that, at each breeding season, many individuals do not bloom and are only found in their vegetative form, which is underground. Thus, at each breeding season, only one sample of the total population is observed, and population sizes can be easily underestimated. The later limitations, likely associated with resource scarcity, may contribute to low levels of genetic variation and increased genetic differentiation between populations [18]. Nevertheless, the relatively low genetic diversity could even facilitate the colonization of endophytic fungi in the fruit of the species. Studies suggest that in mycoheterotrophic species, seeds from cross-pollination have a high frequency of mismatches between plant and fungus, decreasing the germination rate [4]. Thus in *P*. *schenckii* autogamy and low genetic diversity can provide the species an evolutionary advantage that would compensate for the constraints common to mycoheterotrophic plants.

## Conclusions

Mycoheterotrophic species are considered models in studying the interaction between plants and fungi. Due to high morphological, physiological and genomic changes, multidisciplinary approaches are essential to understanding the ecological and evolutionary outcomes of mycoheterotrophy. Our results show mycoheterotrophic species with fungal colonization in aboveground organs. In addition, the absence of pollinators and the prevalence of autogamy have a deeply influence in the genetic structure of this rare mycoheterotrophic plant. Indeed, pollination and seed dispersal occur independently from biotic vectors, supporting the hypothesis of Bidartondo [1], which expects low levels of biological interactions in mycoheterotrophic plants already involved in an obligate symbiotic interaction. Autogamy and dispersal of seeds close to the mother plants contribute to the high levels of genetic structure found in *P. schenckii*; a strategy potentially related to plant-fungal resources, carbon from litter in this case, patchily distributed on the forest understory. Further research is needed to investigate what fungal lineages are involved in plant colonization, if the fungi that provide the initial seed germination is symbiotic, and if there are alternative nutrient sources for the mycoheterotrophic plants, which may indirectly parasite neighboring photosynthetic plants.

## Material and methods

### Plant material and sampling sites

**Fig. 5:**
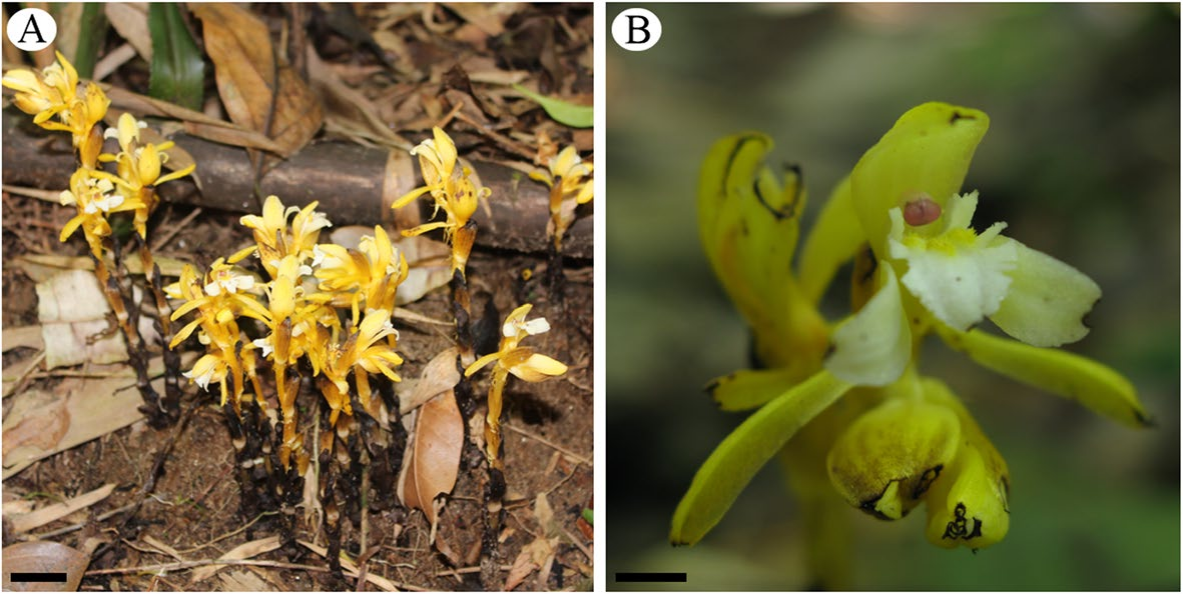
Flowers of *Pogoniopsis schenckii*. **A** Population of *P. schenckii*. **B** Flower in anthesis. Scale bars: 1 cm

*Pogoniopsis schenckii* is an endemic mycoheterotrophic species found in the Brazilian Atlantic Forest. It is a terrestrial plant, achlorophyllous, featuring modified leaves in bracts that cover the stem, flowers with pale yellow color, and indehiscent fruit (Fig. 5a, b). Samples were collected from Parque Estadual da Serra do Mar (Núcleo Santa Virgínia), at the municipality of São Luiz do Paraitinga, São Paulo state, and from the municipality of São Lourenço da Serra, São Paulo state (Supplementary file Fig. S6). The collections were performed from December 2015 to March 2018. After plant sampling, the fungal colonization was studied in axenic conditions. Permits to collect in conservation units were granted by COTEC (Technical and Scientific Committee of the Forestry Institute, São Paulo, Brazil; permission number 597/2014) and SISBIO (Biodiversity Information and Authorization System, Brazil, permission number 44155–6). The formal identification of the samples was carried out by Juliana Lischka Sampaio Mayer. Vouchers were deposited at the Herbarium of the University of Campinas (UEC 196921; 205046).

### Anatomical analyses

In order to analyze the development of reproductive organs, embryogenesis, and fruit development in *P. schenckii*, floral buds were marked and collected from the first to the eighth day after floral opening. In addition, fruits were collected 15, 20, 25, 30, 60, and 90 days after floral opening. The samples were fixed in Karnovsky’s solution [81], dehydrated in serial dilutions of ethanol, and infiltrated with Hydroxyethyl methacrylate [82]. Samples were sectioned at a thickness from 3 to 5 μm using a Leica RM2245 rotary microtome, stained with Toluidine Blue 0.05% in phosphate buffer, pH 4.5 [83], and mounted using Entellan® synthetic resin (Merck®, Darmstadt, Germany). The chemical nature of the substances found in the fruits and embryos were determined using the following histochemical tests: We used test Xylidine Ponceau for proteins [84]; test Ruthenium red for polysaccharides and pectins [85]; test ferric chloride for phenolic compounds; test Sudan III for lipids; and test Lugol for the presence of starch [86]. To verify the presence of fungal hyphae in fruit cells, slides of fruit at different stages of development were doubly stained with Cotton Blue 5% in lactophenol and 1% of aqueous Safranine-O [87]. Slides were analyzed under an Olympus BX51 optical microscope and photographed with an Olympus DP71 digital camera.

### Transmission electron microscopy analyses

In order to analyze the main changes that occur in cells during fruit development, fruits at different developmental stages were fixed in 2.5% glutaraldehyde in 0.2 M sodium cacodylate buffer, pH 7.25, for 24 h [88]. Post-fixation was performed with osmium tetroxide (OsO_4_) 1.0% in sodium cacodylate buffer for 12 h in the dark [89]. The material was dehydrated in a series with increasing concentrations of acetone solution, soaked in LR White® resin (Hard Grade; Fluka), according to the manufacturer’s instructions. Ultrafine sections were prepared with Leica ultramicrotome using a diamond knife. These sections were contrasted with uranyl acetate [90] and lead citrate [91], then examined under a Philips EM 100 transmission electron microscope operated at 80 kV.

### Breeding system, structure and genetic diversity

To verify the occurrence of self-pollination, 10 inflorescences with five floral buds in pre-anthesis were marked, bagged, and monitored until the possible formation of fruits (*n* = 50 in Pirapitinga and Poço do Pito populations). To document the efficiency of natural pollination, 10 inflorescences with five open flowers were marked and not bagged (*n* = 50 in two populations). Flowers used in experimental pollination treatments were monitored until fruit maturation or senescence. Floral visitors were observed with a camera (Sony DCR SR21E). A total of 27 flowers were observed in 17 individuals for 168 h.

A total of 79 individuals from three populations were sampled, two located in Parque Estadual Serra do Mar – Núcleo Santa Virgínia, São Paulo state (Pirapitinga – *n* = 31 and Poço do Pito – *n* = 37), and one located in the municipality of São Lourenço da Serra, São Paulo – *n* = 11 (Supplementary file Fig. S6). DNA extraction was performed using a modified CTAB method following the protocol of [92] (Supplementary file). We selected one individual for Illumina sequencing in order to generate paired-end sequencing reads of 100 bp. These reads were used to search for microsatellite loci and, after testing 20 primers, we found seven polymorphic microsatellite markers that were used here to genotype our sample. Details of sequencing and characterization of microsatellite loci are described in the (Supplementary file). The genetic diversity of each population was estimated using the number of alleles, allelic richness, expected and observed heterozygosity, and the inbreeding coefficient, calculated using the MSA v. 4.05 [93] and HP-RARE v. 1.0 [94] programs. Departures from Hardy–Weinberg equilibrium due to inbreeding within each population were estimated using the GENEPOP v. 4.0 program [95].

To quantify cloning occurrences in populations, three distinct methods were used following the recommendations of Meloni et al. [96]: The ratio between the number of genotypes and total number of individuals in a population (G/N), the Nei diversity index, and the genotypic uniformity index. Values close to zero indicate high clonal propagation values, whereas values close to one indicate sexual reproduction.

The genetic structure of the populations was estimated through pairwise *F*_ST_ and *D*_ST_ among populations, using the ARLEQUIN and GENODIVE [97] programs. The distribution of genetic variation at different hierarchical levels was examined by the analysis of molecular variance (AMOVA) method implemented in the GENODIVE software. The presence of isolation-by-distance was evaluated by the Mantel test, implemented in the GENEPOP v. 4.0 program. The existence of distinct genetic clusters and patterns of genetic mixtures between populations was analyzed by a Bayesian method implemented in the MAVERICK v. 1.0.4 software [98].

## Supplementary Information

**Additional file 1: Figure S1**. Longitudinal and transversal sections of anthers of *Pogoniopsis schenckii*. A. Overview of the tetraesporangiated anther biteca. Arrows indicate the microsporangia. B. Microspore mother cell. C. Dyads of microspores. D. Tetrad of microspores. E. Mature pollen grains, with two cells. F. Release of the mature pollen grain by rupture of the epidermis. G-I. Germination of the pollen grain and arrival of the pollen tube in the placental region. G. Detail of the germination of the pollen grain. H. Detail of the pollen tube reaching the placental region. I. Growth of the pollen tube along the column.; dmi= dyads of microspores; e= epidermis; gc= generative cell; mimc= microspore mother cell p= placental region; pg= pollen grain, pt= pollen tube; tm= tetrad of microspores; vc= vegetative cell. Scale bars: C-F= 20µm, B, H= 50µm, A,G,I= 100µm

**Additional file 2: Figure S2**. Longitudinal sections of ovules of *Pogoniopsis schenckii*. A. General view of the placenta with ovules in the beginning of differentiation. B. Megaspore mother cell. C. Megaspore mother cell during meiosis I. D. Megaspore dyad. E. Calazal megaspore expanding and degenerating micropylar megospores. F. Binucleated megagametophyte. G. Binucleated megagametophyte with nuclei entering the second cycle of mitosis. H. Megagametophyte with three visible nuclei. I. Mature megagametophyte. cm= calazal megaspore; dm= degenerating megaspore; ec= egg cell; md= megaspore dyad; mmc= megaspore mother cell; ngm= nuclei of megagametophyte; p= placental region; pn= polar nuclei; s= synergid. Scale bars: B-I=20µm, A=100µm

**Additional file 3: Figure S3**. Longitudinal sections of ovules and seeds of *Pogoniopsis* *schenckii*. A. Penetrated and non-penetrated synergids. B. Zygote. C. Embryo with two cells. D. Three cell embryo. E. Five cell embryo. F. Six cell embryo. G. Presence of starch as a reserve in the embryo evidenced by Lugol. H. Presence of proteins in the embryo evidenced by Xylidine. ac= apical cell; bc= basal cell; e= embryo; ec= egg cell; f=funiculus; ps= penetrated synergid; s= synergid; z= zygote. Scale bars: A-E,G= 20µm, F,I=50µm

**Additional file 4: Figure S4**. Development of the fruit of *Pogoniopsis* *schenckii. *A, Fruits in different stages of development, with detail of a sectioned fruit. B, Fruit at the beginning of ripening, with detail of a sectioned fruit. C, Ripe fruit. D, Detail of a sectioned ripe fruit. Scale bars= 1cm.

**Additional file 5: Figure S5**. Genetic structure suggested by the Maverick software in *Pogoniopsis* *schenckii* based on eight nuclear microsatellite loci.

**Additional file 6: Figure S6**. Map showing the populations sampled of *Pogoniopsis schenckii*. The distribution map was created using the ArcMap 10.7 software, intellectual property of Esri, used herein under license. In black, the population collected in São Lourenço da Serra, SP. In red populations collected at the Parque Estadual Serra do Mar, Núcleo Santa Virgínia, SP. TPi = Trilha da Pirapitinga; TPP = Trilha do Poço do Pito.

**Additional file 7**

## Data Availability

The datasets used and/or analysed during the current study are available from the corresponding author on reasonable request.
