# Supplementary figures and images for: Reproductive development and genetic structure of the mycoheterotrophic orchid Pogoniopsis schenckii Cogn
Source: BMC Plant Biol. 2021 Jul 12;21:332. doi: 10.1186/s12870-021-03118-y (PMC8276481; doi:10.1186/s12870-021-03118-y)

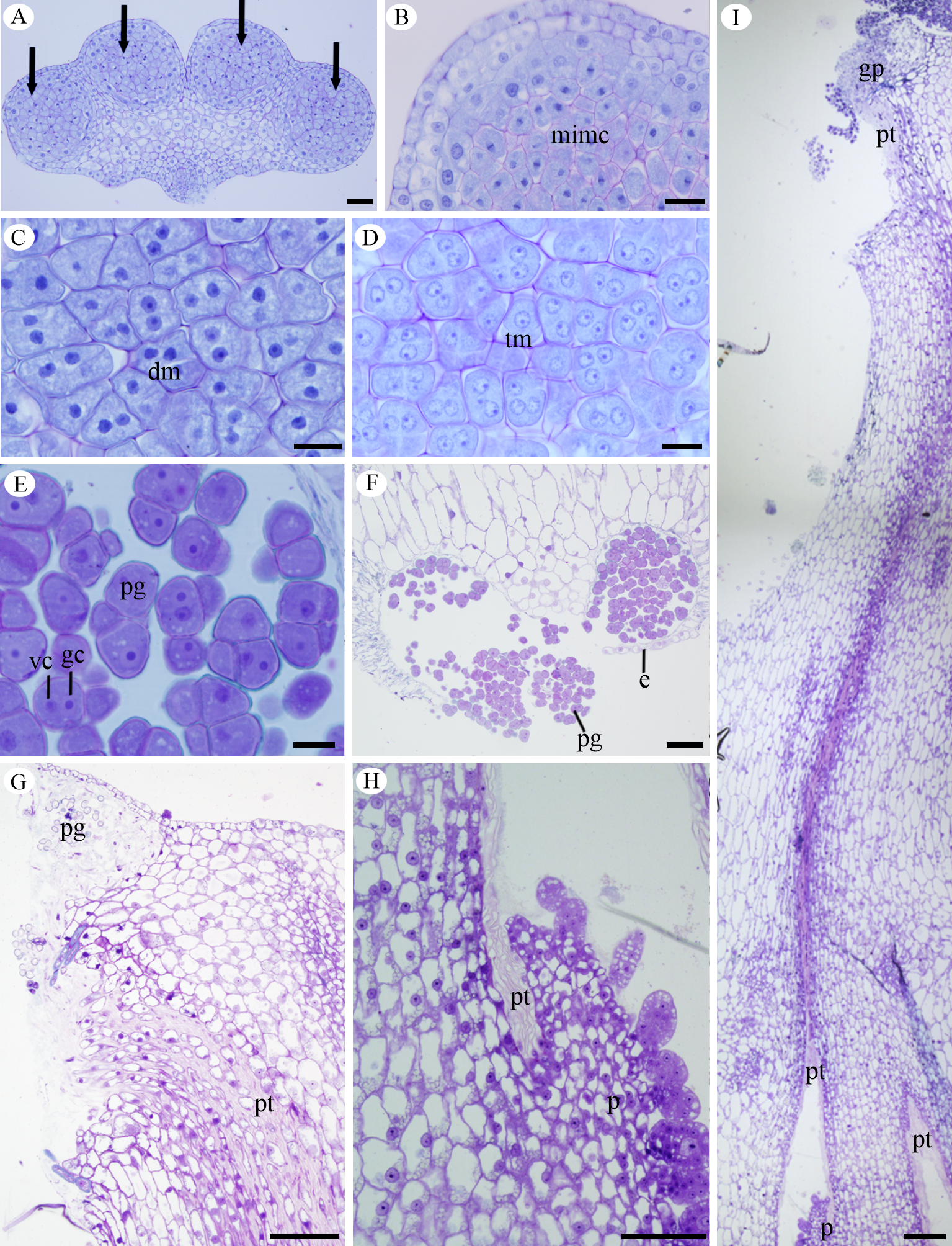

Supplement: Supplementary file 1 — Additional file 1: Figure S1. Longitudinal and transversal sections of anthers of Pogoniopsis schenckii. A. Overview of the tetraesporangiated anther biteca. Arrows indicate the microsporangia. B. Microspore mother cell. C. Dyads of microspores. D. Tetrad of microspores. E. Mature pollen grains, with two cells. F. Release of the mature pollen grain by rupture of the epidermis. G-I. Germination of the pollen grain and arrival of the pollen tube in the placental region. G. Detail of the germination of the pollen grain. H. Detail of the pollen tube reaching the placental region. I. Growth of the pollen tube along the column.; dmi= dyads of microspores; e= epidermis; gc= generative cell; mimc= microspore mother cell p= placental region; pg= pollen grain, pt= pollen tube; tm= tetrad of microspores; vc= vegetative cell. Scale bars: C-F= 20µm, B, H= 50µm, A,G,I= 100µm [file 12870_2021_3118_MOESM1_ESM.tif]

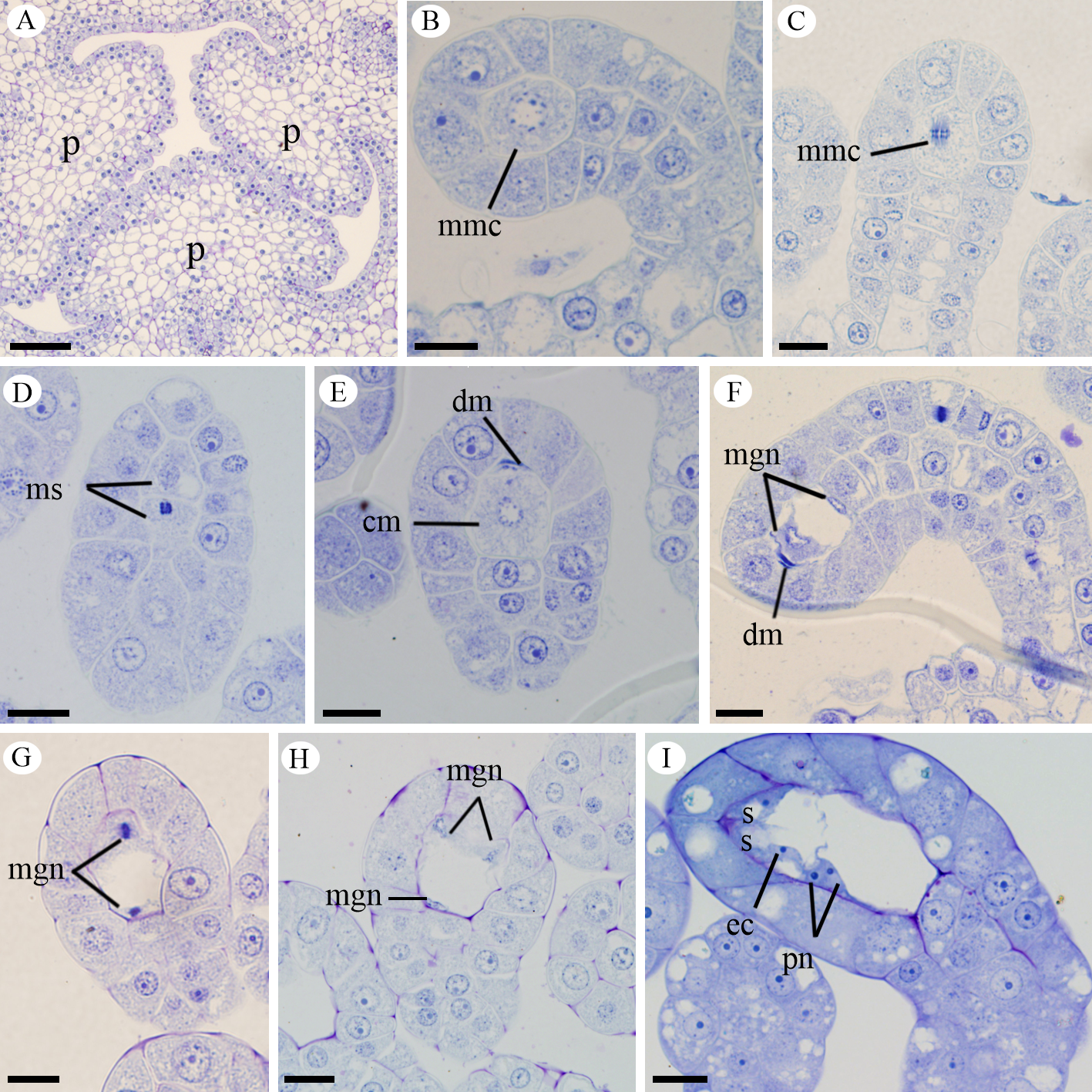

Supplement: Supplementary file 2 — Additional file 2: Figure S2. Longitudinal sections of ovules of Pogoniopsis schenckii. A. General view of the placenta with ovules in the beginning of differentiation. B. Megaspore mother cell. C. Megaspore mother cell during meiosis I. D. Megaspore dyad. E. Calazal megaspore expanding and degenerating micropylar megospores. F. Binucleated megagametophyte. G. Binucleated megagametophyte with nuclei entering the second cycle of mitosis. H. Megagametophyte with three visible nuclei. I. Mature megagametophyte. cm= calazal megaspore; dm= degenerating megaspore; ec= egg cell; md= megaspore dyad; mmc= megaspore mother cell; ngm= nuclei of megagametophyte; p= placental region; pn= polar nuclei; s= synergid. Scale bars: B-I=20µm, A=100µm [file 12870_2021_3118_MOESM2_ESM.tif]

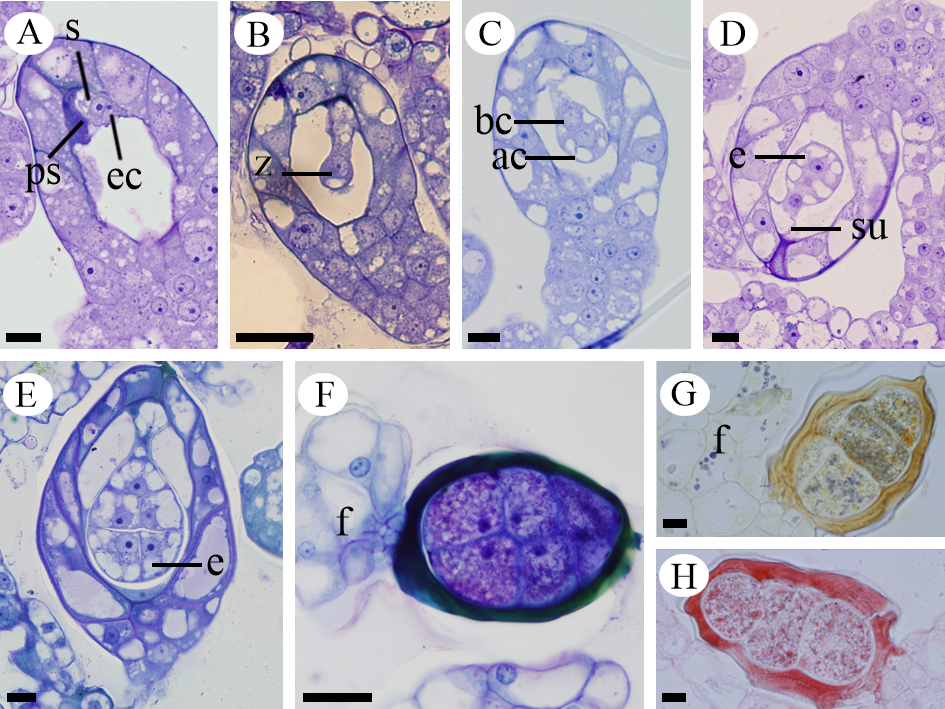

Supplement: Supplementary file 3 — Additional file 3: Figure S3. Longitudinal sections of ovules and seeds of Pogoniopsis schenckii. A. Penetrated and non-penetrated synergids. B. Zygote. C. Embryo with two cells. D. Three cell embryo. E. Five cell embryo. F. Six cell embryo. G. Presence of starch as a reserve in the embryo evidenced by Lugol. H. Presence of proteins in the embryo evidenced by Xylidine. ac= apical cell; bc= basal cell; e= embryo; ec= egg cell; f=funiculus; ps= penetrated synergid; s= synergid; z= zygote. Scale bars: A-E,G= 20µm, F,I=50µm [file 12870_2021_3118_MOESM3_ESM.tif]

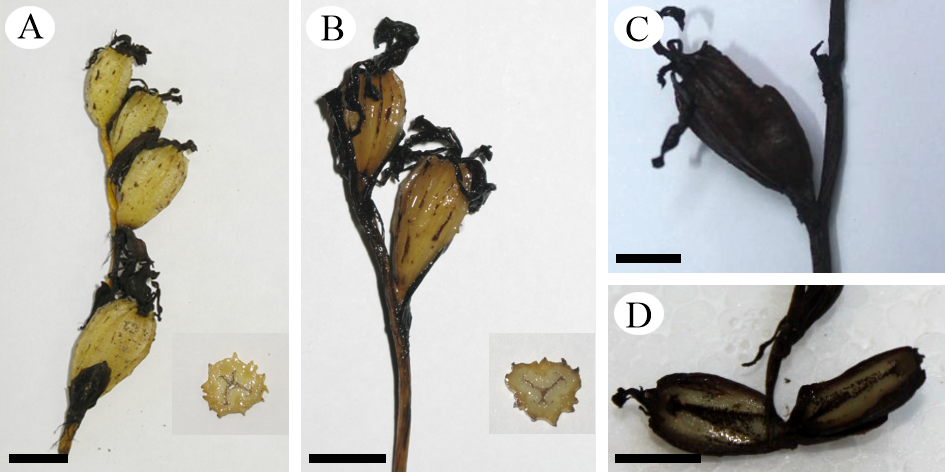

Supplement: Supplementary file 4 — Additional file 4: Figure S4. Development of the fruit of Pogoniopsis schenckii. A, Fruits in different stages of development, with detail of a sectioned fruit. B, Fruit at the beginning of ripening, with detail of a sectioned fruit. C, Ripe fruit. D, Detail of a sectioned ripe fruit. Scale bars= 1cm. [file 12870_2021_3118_MOESM4_ESM.tif]

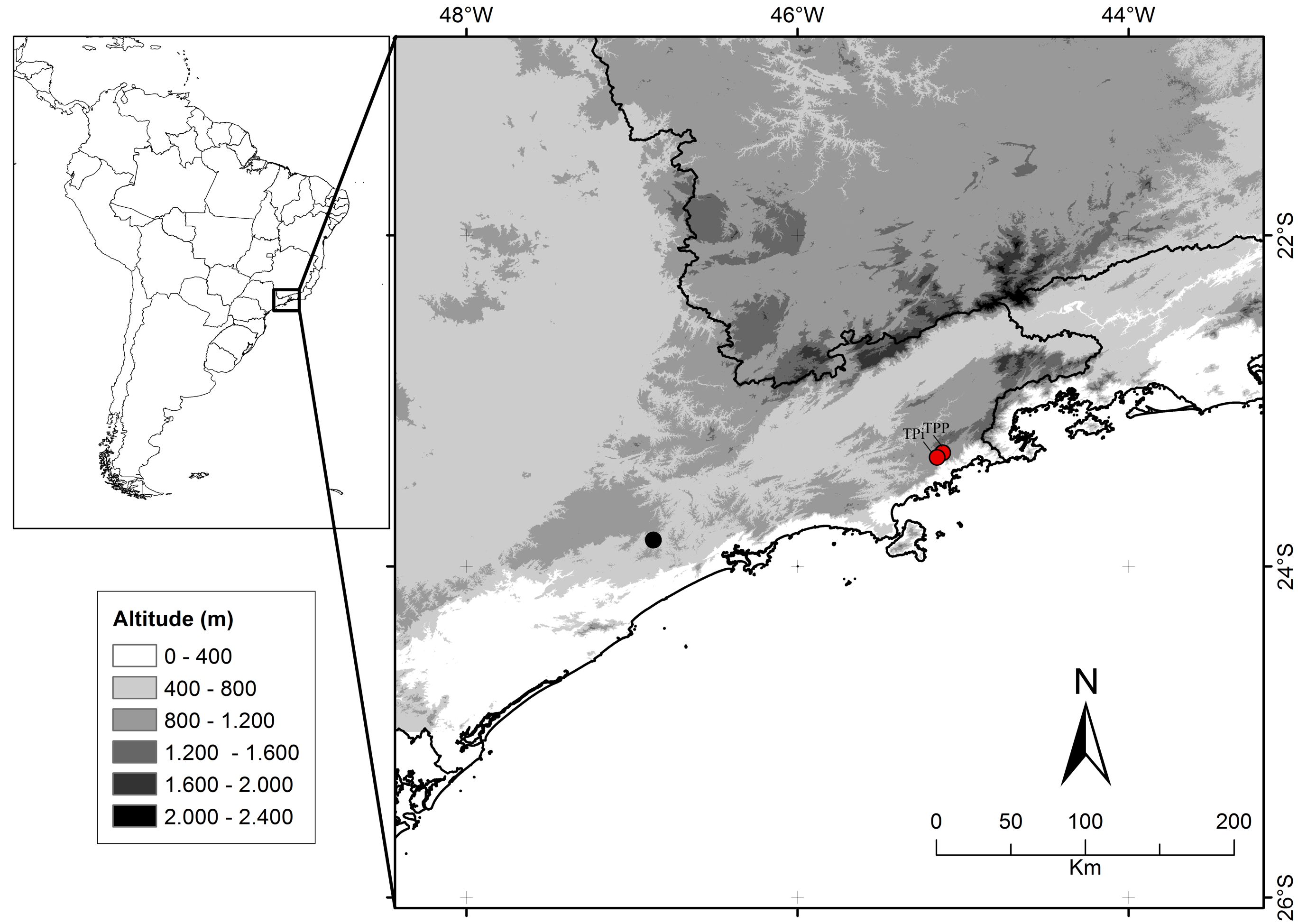

Supplement: Supplementary file 6 — Additional file 6: Figure S6. Map showing the populations sampled of Pogoniopsis schenckii. The distribution map was created using the ArcMap 10.7 software, intellectual property of Esri, used herein under license. In black, the population collected in São Lourenço da Serra, SP. In red populations collected at the Parque Estadual Serra do Mar, Núcleo Santa Virgínia, SP. TPi = Trilha da Pirapitinga; TPP = Trilha do Poço do Pito. [file 12870_2021_3118_MOESM6_ESM.tif]
