# Supplementary material for: Reproductive development and genetic structure of the mycoheterotrophic orchid Pogoniopsis schenckii Cogn
Source: BMC Plant Biol. 2021 Jul 12;21:332. doi: 10.1186/s12870-021-03118-y (PMC8276481; doi:10.1186/s12870-021-03118-y)
Supplement: Supplementary file 7 — Additional file 7 [file 12870_2021_3118_MOESM7_ESM.doc]

**Supplementary Information**

**Isolation and characterization of microsatellites**

DNA extraction was performed from a sample of an individual's fruit, using the CTAB method following the protocol of Doyle and Doyle [1]. The extracted DNA was used to prepare a library for sequencing on the Illumina platform, following the manufacturer's protocol. One tenth of an Illumina lane was used for next-generation sequencing, generating a total of 15,468,621 paired-end reads, with an approximate size of 100 base pairs. The partial genome assembly was performed using the SPAdes program [2] with standard parameters as suggested in the manual. The algorithm assembled the partial genome using a k-mer value of 55 base pairs, producing 270,918 sequences with an N50 of 316 base pairs. The voucher specimen was deposited at the Herbarium of the University of Campinas (HUEC), Campinas, São Paulo, Brazil (registration number UEC 139077).

The Tandem Repeats Finder program [3] was used to filter for the presence of microsatellites in the total set of assemblies obtained (270,918). Only sequences that presented microsatellites after the 30 initial base pairs and contained a number between 8 and 15 repeats were considered into the subsequent analyzes. This resulted in 180 dinucleotides, 127 trinucleotides and 24 tetranucleotides microsatellites found in assembled sequences. For each SSR, the forward primers were synthesized with a 19-foot-long 5′M13 tail (5′-CACGACGTTGTAAAACGAC-3′), and the amplification reactions were performed following Schuelke's [4]. The amplification product underwent sequencing reactions in the Applied Biosystems 2700 thermal cycler in 10 µl of reactions containing: 10 ng DNA model, 1x PCR buffer, 2 mM MgCl2, 100 µl dNTP, 1 pmol primer primer, 4 pmol primer reverse, 0.4 pmol of M13 universal primer and 0.5U of Taq polymerase (Amersham Pharmacia Biotech). For the touchdown cycle program, 95 °C was used for 3 min, then 10 cycles of 94 °C for 30s, 58 °C decreasing to 48 °C at 1 °C per cycle for 30s, 72 °C for 30 s, followed by 40 cycles of 94 °C for 30 s, 48 °C for 30 s, 72 °C for 30 s, followed by a final 20 min extension at 72 °C. Microsatellites were determined on a 3130 DNA Analyzer sequence and were sized with standard LIZ (500) using Genemapper v3.7 software (Applied Biosystems).

Four individuals from the three populations of *P. schenckii* were analyzed to evaluate the polymorphism of the primers. To calculate observed and expected heterozygosity and to test the Hardy-Weinberg Equilibrium output and linkage imbalance between all loci pairs. Arlequin 3.11 was used [5].

Twenty primer pairs were designed, and of these 8 showed clear amplifications (Table 1). Seven markers showed polymorphism and a monomorphism (Pog 14) for the Poço do Pito and São Lourenço populations (Table 1). The amplified products ranged from 129 to 234 bp (Table 1). The number of alleles observed per locus ranging from 2 to 7, and the heterozygosity observed for the polymorphic locus ranged from zero to 0.692 (Table 1).

Primers have proven to be useful in revealing levels of diversity in populations and therefore can be used to explore the diversity, genetic structure and conservation of fragmented populations of *P. schenckii.*

**References**

1. Doyle JJ, Doyle JL. Isolation of plant DNA from fresh tissue. Focus. 1990;12:13-15.

2. Bankevich A, Nurk S, Antipov D, Gurevich AA, Dvorkin M, Kulikov AS, et al. SPAdes: A New Genome Assembly Algorithm and Its Applications to Single-Cell Sequencing. J. Comput. Biol. 2012;19:455–477.

3. Benson G. Tandem repeats finder: a program to analyze DNA sequences. Nucleic Acids Res. 1999;2:573–580.

4. Schuelke M. An economic method for the fluorescent labelling of PCR fragments. Nat. Biotechnol. 2000;18:233–234.

5. Excoffier L, Laval LG, Schneider S. 2005. arlequin, Version 3.0: na integrated software package for population genetics data analysis. Evol. Bioinform. Online. 1990;1:47–50.

Table 1. Characteristics of microsatellite loci from *Pogoniopsis schenckii*, including locus name, primer sequences, repeat types, number of alleles (*A*), observed (*H*O) and expected (*H*E) heterozygosity for each population, and the significance of the test for departure from Hardy–Weinberg equilibrium (HWE). Locus Pog 14 was monomorphic for Poço do Pito and São Lourenço population. GenBank Accession nos. MT274012–MT274019.

| **Locus** | **Primer sequence (5' 3')** | **Repeat** | **Size range (pb)** |  | **Pirapitinga** | | **Poço do Pito** | | **São Lourenço** | |
| --- | --- | --- | --- | --- | --- | --- | --- | --- | --- | --- |
|  |  |  |  | **A** | ***H*O** | ***H*E** | ***H*O** | ***H*E** | ***H*O** | ***H*E** |
| Pog 06 | F ATTTTTCGCTGTCCGACCT | (AT)14 | 150 | 7 | 0,032 | 0,47 | 0,405 | 0,637 | 0 | 0,415* |
|  | R TCACTCTGTAAAGGAATCAGC |  |  |  |  |  |  |  |  |  |
| Pog 08 | F CGACAGGTTCGAAGAGTTT | (AT)13 | 155 | 3 | 0,580 | 0,621 | 0,648 | 0,672 | 0,818 | 0,506* |
|  | R AAATACGCTCCAAAACACAC |  |  |  |  |  |  |  |  |  |
| Pog 11 | F GGCTTTCATCGTATCTTGTG | (AT)14 | 148 | 6 | 0,387 | 0,634 | 0,27 | 0,665 | 0,181 | 0,593 |
|  | R TCATCGTGTTTGATTGAGC |  |  |  |  |  |  |  |  |  |
| Pog 14 | F GGCTATTTGCAATGGAGTAA | (AT)12 | 139 | 2 | 0 | 0,122 | mono | mono | mono | mono |
|  | R TAATTGTATCGTGAGCAACG |  |  |  |  |  |  |  |  |  |
| Pog 16 | F GCCGTTTAATATCCGTACC | (AT)12 | 234 | 5 | 0 | 0,404 | 0 | 0,654 | 0,090 | 0,558 |
|  | R TAATCTTTGCTGCGTTGAC |  |  |  |  |  |  |  |  |  |
| Pog 24 | F TTAGCAACGAGAATGGAAGT | (TA)13 | 129 | 5 | 0 | 0,283 | 0,567 | 0,635 | 0 | 0,329* |
|  | R ATCCACTATATGTCGCATGG |  |  |  |  |  |  |  |  |  |
| Pog 25 | F AGACACTGCATCACATGAAA | (TA)13 | 156 | 5 | 0,354 | 0,484 | 0,567 | 0,692 | 0,636 | 0,688 |
|  | R CGCGTGTCGTGTGTACTAT |  |  |  |  |  |  |  |  |  |
| Pog 32 | F CCAGGGTTTTAAGTGCATAC | (AT)14 | 161 | 7 | 0,225 | 0,405* | 0,351 | 0,663 | 0,181 | 0,173* |
|  | R GCAGAGCTGCTACAAGTAAAG | |  |  |  |  |  |  |  |  |

*Significant departures from HWE: P<0,001
